# Production of Starch-Based Flexible Food Packaging in Developing Countries: Analysis of the Processes, Challenges, and Requirements

**DOI:** 10.3390/foods13244096

**Published:** 2024-12-18

**Authors:** Johanna Garavito, Clara P. Peña-Venegas, Diego A. Castellanos

**Affiliations:** 1 Food Packaging and Shelf Life Laboratory, Instituto de Ciencia y Tecnología de Alimentos, Universidad Nacional de Colombia, Carrera 30 Número 45-03, Edificio 500A, Bogotá 111321, Colombia; ngaravitoj@unal.edu.co; 2 Instituto Amazónico de Investigaciones Científicas—SINCHI, Avenida Vásquez Cobo Calle 15/16, Leticia 910001, Colombia; cpena@sinchi.org.co

**Keywords:** bio-packaging, sustainable process, biobased materials, single-use, scaling up

## Abstract

Biodegradable packaging offers an affordable and sustainable solution to global pollution, particularly in developing countries with limited recycling infrastructure. Starch is well suited to develop biodegradable packages for foods due to its wide availability and simple, low-tech production process. Although the development of starch-based packaging is well documented, most studies focus on the laboratory stages of formulation and plasticization, leaving gaps in understanding key phases such as raw material conditioning, industrial-scale molding, post-production processes, and storage. This work evaluates the value chain of starch-based packaging in developing countries. It addresses the challenges, equipment, and process conditions at each stage, highlighting the critical role of moisture resistance in the final product’s functionality. A particular focus is placed on replacing single-use plastic packaging, which dominates food industries in regions with agricultural economies and rich biodiversity. A comprehensive analysis of starch-based packaging production, with a detailed understanding of each stage and the overall process, should contribute to the development of more sustainable and scalable solutions, particularly for the replacement of single-use packages, helping to protect vulnerable biodiverse regions from the growing impact of plastic waste.

## 1. Introduction

The need to reduce the pollution generated by the large-scale production of plastic packaging has become an urgent global goal [1]. Countries are implementing regulations to promote the mandatory replacement of traditional non-biodegradable plastics with sustainable alternatives in the short and medium terms. While traditional cellulose-based materials like paper and cardboard dominate the food packaging market [2], alternatives such as polylactic acid (PLA), polybutylene succinate (PBS), polyhydroxyalkanoates (PHA), polybutylene adipate terephthalate (PBAT), and starch have gained increasing attention globally [3,4]. Despite these efforts, many alternatives have not yet fully replicated the cost-efficiency, scalability, and environmental benefits expected globally, especially in developing regions, where infrastructure limitations and economic constraints challenge their adoption. For single-use flexible packaging, the main goal is to obtain products comparable to traditional petrochemical polymers, combining lightness, durability, versatility, and cost-effectiveness [5]. Starch-based packaging has shown promise for preserving the quality of perishable foods such as fruits, vegetables, and bakery products by providing a biodegradable barrier against moisture and oxygen [6]. This potential to reduce spoilage and extend shelf life makes it especially relevant for food packaging applications in regions with high agricultural productivity but limited access to advanced technologies. We searched the keywords “Abstract: (‘starch packaging’ OR ‘starch film’)” in the patent databases (The Lens) and “TITLE-ABS-KEY (‘starch packaging’ OR ‘starch films’)” in the Scopus database of scientific publications and filtered the results between 2022 and 2024, resulting in 103 active patents and 379 scientific documents addressing the methods and processes for flexible packing production. Nonetheless, this patent and research concentration does not reflect global innovation equally, and a significant geographical bias exists: 56% of active patents corresponded to the USA, 33% to China, and the remaining to Europe, South Africa, and Australia. Regarding academic production, 75% of publications corresponded to Asian countries, North America, and Europe, and only around 25% of the publications stemmed from South America (Brazil significantly, with more information corresponding to 12% of publications) and Africa. This imbalance is not trivial; it suggests that important innovations required for regions with unique challenges (such as limited industrial capacity or reliance on local, natural materials) may be underexplored. Latin America holds significant potential for biodegradable packaging innovation due to its high biodiversity and low labor costs, but technical and industrial barriers often hinder this potential. The literature focuses primarily on specific stages of the process, such as the formulation of thermoplastic starch (TPS) to address mechanical deficiencies [7,8] and the plasticization or molding processes to improve product viability [9,10]. However, this segmented focus fails to address the scalability and commercialization challenges of low-resource settings.

This review was conducted using a systematic approach to ensure relevance, rigor, and comprehensive coverage of the subject. The research was sourced from databases such as Scopus, Elsevier, Google Scholar, Web of Science, and The Lens (for patents), focusing on advancements in starch-based packaging. The search employed Boolean operators and keywords such as “starch-based packaging” AND “biodegradable materials” AND “developing countries” and “starch extrusion” AND “sustainable packaging technologies”, initially retrieving 250 scientific articles and patents. The inclusion criteria prioritized peer-reviewed studies, patents, and publications from 2020 to 2024 for recent advancements, while foundational works before 2019 provided essential context. Each section of the article was designed to address specific stages of the production process, using targeted keywords relevant to the concepts discussed, such as material reinforcement, available technologies, and their implications for scalability.

This article aimed to synthesize existing documentation to present a complete production process, analyzing the implications of each stage—from raw material sourcing to post-production. This required integrating technical concepts, foundational pillars, and recent advancements to ensure a cohesive narrative that connects critical points, such as the role of material reinforcement, the impact of emerging technologies, and the challenges of scaling in resource-limited settings. By combining recent research with established principles, this review offers a structured framework for understanding the production and application of starch-based packaging, addressing both theoretical insights and practical solutions.

## 2. Flexible Bio-Packaging Overview

**Figure 1:**
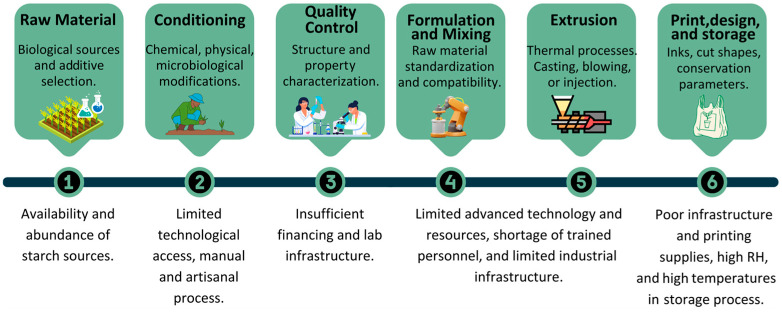
Production stages of starch-based flexible packaging and considerations for developing countries.

Environmental and economic pressures undoubtedly drive the global transition from petrochemical-based single-use packaging to biodegradable alternatives. Policies such as Directive (EU) 2019/904 aim to reduce single-use plastics by 2026, addressing the 60% of plastic waste these materials contribute in the EU [11]. However, merely implementing regulatory frameworks is insufficient to close the global gap between ambitious environmental goals and practical solutions. Although plastic recycling plays a crucial role in waste management, there are notable disparities in recycling capacities between developed and developing countries. For instance, while regions like North America, Europe, and China recycled approximately 33 million tons of plastic waste in 2019, Latin America and Africa recycled only 5.5 million tons, highlighting the need for alternative strategies better suited to these contexts [12]. It is suggested that for developing countries, it will be more feasible, practical, and cheaper to produce bio-packages to replace plastics than develop recycling infrastructure in regions with low recycling education, difficult road access, low industrial development, poor urban infrastructure, and poor selective waste collection [13,14]. Furthermore, many biodegradable materials available on the global market, such as PLA and PBAT or even biomaterials made from modified starches, are rarely accessible to developing countries due to high import costs and tariffs, which make them uncompetitive in local markets. This creates a significant barrier to their widespread use in these regions. The production of flexible starch-based packaging involves multiple stages, including raw material obtainment, conditioning, characterization, formulation, extrusion, molding, printing, packaging, and storage, each tailored to address specific challenges (Figure 1).

Low-cost methods include solvent casting, where a polymer solution is spread on a substrate and the solvent is removed. This process includes the possibility of incorporating active agents into the resulting films [15]. Tape-casting is a similar technique in which an adjustable blade is used to dry the mixture on a moving belt and control the thickness, forming a uniform film [16]. In both cases, plasticizers are essential for the steady formation of the film [17]. Nevertheless, while low-cost techniques like these may seem ideal, they often result in films with poor mechanical strength and water resistance, making them less suitable for practical applications in food packaging. Other techniques use heat and pressure to convert starch into a stronger plastic material with lower water vapor permeability, such as compression molding [18] or blown film extrusion. These techniques require high mechanical strength due to the tension generated during blowing [19], so are blended with PBTA, celluloses [20], or nano clay reinforcements [21] to enhance the biofilm’s properties. Flat sheets produced through extrusion are used in thermoforming applications, where adequate moisture resistance is essential to preserve their structural integrity post-molding. It has been shown that adding a reinforcement of up to 15% fiber in a starch-based formulation decreased the moisture absorption rate but was insufficient to retain liquid products such as those exuded by fresh foods [22]. So, hydrophobicity is typically achieved through chemical modifications such as hydroxypropylation [23] or the incorporation of complex mixtures, including combinations of nanofillers, clays, resins from polymers like PLA or PBAT, natural waxes, and cellulose derivatives [24]. The improvement of hydrophobicity in starch-based materials can also be achieved through the incorporation of hydrophobic coatings and coupling agents such as (3-aminopropyl) triethoxysilane (APTES), which promote the formation of dynamic cross-linked networks between hydrophilic and hydrophobic layers [25]. Improving bioplastics’ mechanical strength, gas permeability, and moisture resistance remains critical for their adoption in food packaging. Recent innovations, including biocomposites and functional chemical modifications, have enhanced laboratory-scale prototypes [7,26] or chemical modifications, introducing functional groups [27].

Even so, scalability issues persist due to the inconsistency of raw materials and the lack of standardized production methods in developing regions. Packaging is particularly relevant for the food industry, where sustainability and safety are paramount. Starch is biodegradable, cost-effective, and easily combined with organic and inorganic reinforcements. Compared to cellulose or other bioplastics, starch-based solutions require less complex infrastructure, making them suitable for small-scale production in developing countries [28]. In Colombia and Brazil, farming communities have demonstrated the effectiveness of rudimentary methods to extract starch from different species with efficiencies of around 33.5–41% [29]. However, physical starch contamination due to the need for industrial equipment might affect its commercial acceptance [30].

## 3. Procurement and Preparation of Raw Materials

In addition to its recent applications in the production of flexible packaging materials, starch has traditionally been used in the food industry as a texturizing agent [31] and in soups, dressings, and sauces [32]. Starch sources determine the viscosity, thermal stability, and retrogradation [33]. Starch has recently gained significant attention as a raw material for non-food applications, with approximately 27% of roots and tubers allocated for biofuel production and industrial processing [34]. Starch is commonly obtained from tubers, roots, cereals, and fruits, depending on geographical location, climate, and tradition [35]. Corn, potato, cassava, wheat, and rice are the most common worldwide starch sources. In 2020, world starch production exceeded 90 million tons, with 95% produced in Asia, Europe, and the United States and only 3.4% in Latin America [31]. Despite the global increase in production, developing countries face limited access to industrial-grade starch, creating a competitive disadvantage in local markets. Additionally, balancing industrial applications with starch’s role as a staple food is critical in regions where food security is a concern. Diverting edible to industrial uses can strain local food systems. To address this, there is growing interest in exploring non-edible starch sources and agro–industrial residues, such as potato or cassava peels, as alternatives for bio-packaging production [36]. These by-products, often discarded as waste, offer a sustainable alternative for bioplastics without compromising food supplies while contributing to circular economic initiatives by reducing waste and generating value from unused materials. Brazil is the largest starch producer in Latin America, ranked 16th in global exports with USD 76.7 million in 2022 [37]. Colombia produced approximately 6.4 million tons of starch in 2019, of which 48.8% was potato starch [38]. This unequal access to industrial starch sources limits the ability of developing countries to fully integrate starch-based bioplastics into their production chains, reinforcing the need for localized starch extraction and production methods that do not compromise food security.

### 3.1. Types of Starch and Characteristics

Starch is a polysaccharide composed of glucose units arranged in two main structures: amylose and amylopectin. The ratio between these molecules, structure, and molecular dimensions varies by origin [39] (Table 1), directly affecting the properties of thermoplastic starch (TPS). Starches high in amylose offer greater resistance, hardness, and crystallization, while those rich in amylopectin enhance flexibility [40]. However, this simplistic correlation between starch composition and TPS properties overlooks real-world complexities, where interactions with plasticizers, environmental factors (e.g., humidity), and chain mobility significantly affect material performance, making it necessary to tailor starch formulations for specific uses [41].

Amylose tends to be more hydrophilic than amylopectin due to its structure, which increases the exposure of hydroxyl (-OH) groups. While these groups are chemically identical in both molecules, the structural linearity of amylose enhances its interaction with water molecules, leading to dimensional instability, increased water permeation, and reduced tensile strength when applied in packaging systems. To mitigate these effects, techniques such as nano-reinforcement [49], hybridization, and cross-linking methods [50] are often suggested to mitigate these mechanical issues; their practical implementation can be costly and may introduce other challenges, such as decreased biodegradability or increased material stiffness. The amylose/amylopectin ratio significantly influences starch permeability to water, lipids, and gases [51]. Amylose is formed of α-1,4 glycosidic bonds that allow for more compact structures than amylopectin, making it less permeable to gases [52,53]. This lower permeability comes at the cost of flexibility, a trade-off that needs to be critically considered depending on the application. For example, while lower gas permeability may benefit certain types of packaging, it may reduce the material’s versatility in applications requiring flexibility and durability in dynamic environments.

The amylose and amylopectin ratio can influence the color and opacity of starch-based biopolymers. Higher amylose content results in greater transparency and lighter color due to its linear structure, while the branched structure of amylopectin scatters light, reducing transparency [54]. This highlights the need to tailor visual and functional properties to the specific application, as excessive transparency may not suit all packaging needs. Starch-based biopolymers are highly biodegradable [55] but their degradability and solubility depend on the amylose/amylopectin ratio [56]. Higher amylose content increases crystallinity, slowing decomposition, which can enhance durability but may not align with short-term packaging needs like those for food [57]. While slower degradation might benefit packaging durability, it could present a challenge in applications with rapid biodegradability, such as short-term food packaging, which is essential. Thus, balancing durability with environmental impact remains a consideration.

### 3.2. Raw Material Extraction and Conditioning Processes

Starch extraction and conditioning processes vary in complexity according to the starch source, the scale of production, and the technology used. Industrial methods are essential for ensuring starch purity, uniformity, and quality required for food-grade applications and bioplastics. Tubers and roots undergo washing, peeling, and grinding to create an aqueous starch solution, purified through continuous washing and centrifugation to remove impurities, fibers, and proteins [58,59]. Starch granules are then dehydrated using hot air drying, sunlight exposure, or spray drying to achieve optimal moisture levels for preservation [60]. Maintaining controlled humidity during storage, particularly for food packaging applications, prevents hydration/condensation and microbiological instability. For instance, cassava flour requires humidity below 9% to ensure safety and reduce hygroscopicity [61]. Uniform particle size achieved through grinding and sieving is critical for ensuring consistent bioplastic properties during extrusion.

In developing countries, starch production is often artisanal, deeply rooted in ancestral traditions with some technological improvements over time, such as graters, sieves, and dryers [62]. These methods typically produce small volumes with inconsistent quality, which is insufficient for industrial bioplastics [59,63]. Starch separation is performed manually using filter fabrics, obtaining a moist material with a starch content of around 60–70% [58], which is adequate for food preparations [64]. In cases requiring a more dehydrated starch, artisanal dehydration is used by exposing the material to sunlight or into a drier environment, placing thin layers of starch on flat surfaces and usually covering them with a dark plastic film to increase the water evaporation, obtaining a compact starch with around 10 to 15% of humidity [58]. This process is usually long and reduces the quality of the starch, and depending on the weather conditions, it may only be possible to reduce the humidity by 20 to 30% [63]. Starch granules must have a moisture content between 8% and 15% during extrusion to secure a thermoplastic transition and a higher expansion index without the erratic viscosity increase [65]. Starches with high amylose content have more humidity and require higher temperatures for thermoplastic transition [66]. To overcome these challenges, cooperatives or small producer associations could pool resources to increase starch volumes and improve local economies, fostering fair trade and a reliable supply chain.

Incorporating locally sourced and innovative raw materials offers sustainable alternatives for starch-based bioplastics in developing countries. Examples include cellulose nanofibers from sugarcane bagasse [67,68]; glycerol, derived from biodiesel production, which is an economical and biodegradable plasticizer; and castor oil, a natural plasticizer, which improves the flexibility and durability of starch-based bags [69,70]. Agricultural by-products, such as natural reinforcements like coconut fiber and chitin [71], as well as pineapple fiber [72], have enhanced the mechanical strength of starch bags without compromising their biodegradability. Non-edible starch from agro–industrial wastes [73] and native seeds such as avocado, sorghum, quinoa, and mango [74] have also been investigated, providing starch and fiber that can produce biopolymers, promoting sustainable new industries. These materials reduce production costs, promote the circular economy, and enable the development of new industries while maintaining biodegradability and functionality.

### 3.3. Transportation of Raw Starch

The storage and transportation of hydrophilic starch with a high moisture retention capacity (type II moisture sorption isotherms and high solubility) [75], requires controlled conditions that guarantee its characteristics until application. Starch should be stored with a moisture content between 0.2 and 0.6 to prevent fermentative or enzymatic deterioration, which could compromise its quality [76]. Cool, dry environments with minimal temperature and humidity fluctuations are essential, and airtight containers are recommended for storage [77]. Industrial processes often rely on advanced technologies, such as air conditioning, dehumidifiers, and hermetically sealed silos, to maintain these conditions. Transport vehicles with controlled environments further ensure starch quality during transit [78]. In developing countries, implementing these controlled environments is often prohibitively expensive. Energy-intensive technologies, such as dehumidifiers, are inaccessible to regions where energy use is costly, and where 35% of the population still relies on firewood for cooking [79]. The lack of infrastructure limits the scalability of starch production for bioplastic applications, as inadequate storage and transportation lead to quality deterioration before use. Addressing these gaps is crucial to expanding starch-based bioplastic production in these regions by developing cost-effective storage and transportation solutions tailored to local contexts.

### 3.4. Formulation

Due to its unique physicochemical properties, starch processing is more complex than other synthetic or natural polymers. Starch biofilms can be fragile and have limited mechanical strength. To overcome these critical points, additives such as metal oxide nanoparticles [80] or essential oils can improve properties such as permeability, hydrophobicity, stability, and UV protection [81]. Despite these improvements, reliance on synthetic additives, while effective in developed regions, risks undermining starch’s core advantage: biodegradability. Plasticizers, emulsifiers, and cross-linkers like epichlorohydrin and phosphorus oxychloride improve hydrophobicity and form stable three-dimensional structures [82]. Still, their environmental cost cannot be overlooked, so the focus must shift to sustainable, locally available alternatives. Addressing degradation control remains critical. While nanofillers like cellulose enhance biodegradability, fullerenes and carbon nanotubes, though effective in enhancing durability, reduce starch’s natural decomposition [83]. Instead of relying on these, developing nations should prioritize natural nanofillers like chitosan [84], which maintain durability and biodegradability, ensuring that starch-based packaging aligns with economic and environmental goals.

### 3.5. Additives

Functional compounds play a specific role in the formulation of bioplastics (Table 2). These compounds enhance the material’s properties, such as flexibility, durability, and processing stability, enabling bioplastics to compete with conventional plastics across various applications.

### 3.6. Reinforcing Materials

Reinforcement materials enhance starch-based polymers’ mechanical strength, thermal stability, and barrier properties. These include cellulose fibers, natural fibers (hemp, jute, sisal, cotton, bamboo, bagasse), clays, fatty agents (waxes, paraffins), and agro–industrial residues such as potato and cassava peels. Cellulose and natural fibers provide rigidity and structural strength, clays improve gas barrier and thermal resistance, and fatty agents enhance water resistance and vapor barrier properties (Table 3). In developing countries, conventional reinforcements’ high cost and limited availability drive interest in locally sourced alternatives. Research studies are increasingly focused on materials derived from agro–industrial waste, food production by-products, or abundant local clays, which are renewable and widely available [98]. Some identified reinforcing materials include hemp, jute, sisal, cotton, wood, bamboo, and bagasse [99]. These materials reduce production costs, lessen import dependence, and promote environmental sustainability. This approach supports bio-packaging innovation by addressing challenges like improving polymer compatibility, achieving uniform dispersion, and tailoring properties for diverse applications, contributing to a circular economy by transforming waste into valuable resources.

## 4. Mixing, Plasticizing, and Pelletizing

### 4.1. Mixing and Plasticizing

Mixing combines starch, additives, and reinforcing compounds to create a uniform biofilm matrix. Uniform dispersion is crucial for consistent properties but can be challenging with equipment or technological limitations [107]. The mixing temperature and speed play critical roles in ensuring proper dispersion. Temperatures between 15 and 50 °C are optimal; lower temperatures hinder liquid absorption, while higher temperatures (above 60–80 °C) risk premature gelatinization, causing granule swelling, loss of crystallinity, and amylose leaching [108]. Speed also affects the mixture’s viscosity and texture; excessive speeds increase density but reduce uniformity, as demonstrated in studies with “Sagu” starch and micro cellulose [109]. High-speed mixers are recommended for TPS/PBSA/PLA combinations, as they improve dispersion before extrusion [110]. The mixing time must be determined for each formulation and process and be enough to disperse the compounds into the mixture, generating a uniform product. The time will depend on the capacity and rpm of the mixing equipment. Considering that speed increases, the final torque decreases, and the final temperature of the mix increases linearly [111]. Because starch retains moisture that bonds molecularly with additives, standing may be necessary after mixing to ensure interaction between all components and allow for proper moisture absorption of the starch granules, depending on the formulation and the subsequent processes applied [112].

Plasticization occurs when the homogeneous mixture is exposed to high temperatures and pressures to melt and soften it, making it malleable and interacting highly with the polymer matrix, plasticizers, and additives. Temperature and pressure control is important during plasticization to avoid starch degradation or other heat-sensitive components [113]. Starches become unstable at temperatures above 180 °C and can only tolerate temperatures up to 200 °C for short periods. The recommended temperatures are between 100 and 150 °C [114,115].

### 4.2. Obtention of Pellets by Extrusion

**Figure 2:**
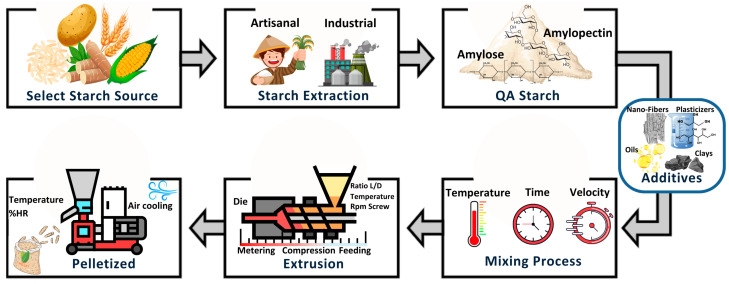
Mixing and extrusion–pelletization process for TPS.

Figure 2 summarizes the sequential steps in transforming raw materials into pellets, highlighting the close relationship between mixing and extrusion techniques and the resulting starch-based bioplastic product.

The extrusion process transforms the raw starch and additives mixture into a thermoplastic starch (TPS) in a high-temperature and dynamic pressure process. The extruder screw provides mechanical energy to melt the starch, mix the additives, and push the material through a die [116]. A pellet is obtained by extruding the polymer matrix through high-temperature molding, where the mixture is plasticized until it becomes a melted material that then goes through a suitable mold to create thin strings, usually with different diameters and sizes (generally between 2 and 4 mm in diameter) [117]. This process occurs in a heated barrel with multiple zones: feed, transition, and metering, ensuring a gradual transformation from a heterogeneous mixture to a homogeneous melted material [118]. Twin screw extruders are often recommended for starch-based materials due to their superior mixing capabilities and ability to handle low-fluidity blends. These extruders can operate with independent feeding systems, allowing for better control of complex mixtures [119]. Since starch tends to form dust, the hopper must have a system that breaks up material agglomerations and allows the material to fall constantly into the extrusion zone. The feeding zone has the lower temperature of the entire barrel of the extruder (usually 90–120 °C for starch) [120] to prevent early gelatinization of the mixture, which can cause strong adhesion to the surface and obstruction of the feeding entrance [121]. Once the material enters into the screws, it melts, flows, and mixes during its passage through the barrel and is pushed forward, generating the mixture’s plasticization. Due to the low fluidity of starch blends, twin screw extruders are recommended for better mixing and flow. Screw speed may vary depending on specific equipment, but frequently between 40 and 120 rpm [122]. The speed must be set according to residence time inside the barrel for the complete mixture plasticization without thermal degradation of any of its components [123]. In the transition section, the fusion of the material occurs and is pumped to the dosing section, where the material is forced to come out through a die with circular holes up to 10 mm in diameter, creating thin strings. Like plastic, these strings must be adequately cooled to solidify before being cut. In the case of starch, this process is carried out using forced convection with cold and dry air due to its low tolerance to moisture. Cutting the pellets must also be performed in dry air, unlike traditional plastic-cutting systems that operate submerged in water to cut and cool the pellets [124].

The equipment required for the extrusion and pelletization processes is usually expensive due to the complexity of the process. In the case of processing starch-based materials, the constant presence of humidity can accelerate oxidation processes in metal parts. An industrial twin screw extruder, depending on its working capacity, can cost between USD 80,000 and 350,000 when purchased from European and American manufacturers such as MAS (Maschinen—und Anlagenbau Schulz GmbH, Pucking, Austria), Hamar Laser (Hamar Laser Instruments, Inc., Danbury, CT, USA), and Brabender (Brabender^®^ GmbH & Co., KG, Germany), while equipment purchased from Chinese manufacturers (e.g., Jiangyin Junzhuo Machinery Manufacturing Co., Ltd., Jiangyin City, China) ranges from USD 30,000 to 100,000. Complementary equipment such as mills, cutters–pelletizers, and mixers also have significant costs ranging between USD 3000 and 30,000, which constitutes a substantial obstacle to the industrial production of starch-based packaging in developing countries and at the global level. For non-industrialized countries, a viable solution is the local production of this type of machinery. In Colombia, for example, small and medium-scale companies can assemble industrial-capacity extruder machines and associated equipment at more affordable prices than imported machinery (USD 10,000–50,000), making starch-based packaging feasible.

## 5. Extrusion and Post-Production

Starch-based bioplastics hold great potential for producing bag-type packaging applicable across the food, cosmetics, and pharmaceutical industries [125]. Despite this, their susceptibility to water vapor and moisture is a notable constraint (even when adding reinforcing materials), especially for liquids or products with high water content, limiting their adoption across diverse industries. While extrusion processes like flat sheet and blow extrusion are effective, they require precise control and sophisticated equipment that can be costly [126]. To overcome this, using locally manufactured extruders, simpler technology, or renewable energy sources like solar power can reduce costs and improve access to starch-based bioplastics production.

### 5.1. Flat Sheet Extrusion Bags

Obtention of bags from flat sheets starts by adding TPS pellets or granules into a sheet extruder machine. The material is homogenized, melted, and shaped by a die that determines the sheet’s width, shape, and thickness [118]. Depending on the biopolymer composition, thickness typically ranges from 0.05 mm to 0.5 mm, with corn, cassava, and wheat starch films commonly measuring between 0.06 mm and 0.22 mm [127]. These dimensions are critical for determining mechanical properties such as tension, flexibility, impact resistance, and hardness, which are influenced by biofilm thickness [128,129]. After extrusion, the sheet undergoes calendering, where rollers improve uniformity and thickness. The process may involve stretching the sheet in one direction or biaxially, enhancing mechanical strength and barrier properties [130]. Once calendering is complete, the sheet is cooled by forced air convection and cut into the desired shapes. Sheets are then thermally sealed at temperatures between 85 and 166 °C to ensure a secure and durable bond [131]. Flat-sheet extrusion is particularly suitable for areas with high humidity, where blow extrusion processes may compromise material strength. While this method involves sealing two sheets, increasing labor requirements, the equipment is generally less expensive and easier to implement, making it an accessible option for resource-limited settings.

### 5.2. Blown Extrusion for Bag Making

In blow molding, the melted material is extruded through an air die head to form a tubular film that expands into a bubble with the help of a forming ring and compressed air [132]. To achieve uniform material distribution and bubble stability, extrusion conditions such as cylinder temperature, screw rotation speed, and extrusion pressure must be optimized. Starch-based films with plasticizers typically operate within a temperature range of 60 to 120 °C [133], but the addition of SiO_2_ can extend this range to 120 to 160 °C [134]. Screw rotation speeds usually fall between 50 and 150 rpm, depending on the mixture composition. Higher speeds can promote material bonding, while lower speeds may be insufficient for effective starch processing [120]. For mixtures with glycerol, 100 rpm is adequate, whereas 40 rpm is more suitable for those containing urea and formamide as plasticizers [135]. However, these parameters can vary based on screw design and equipment. Advances in incorporating carboxymethyl chitosan (CMCS) and optimizing PVA polymerization have significantly improved starch-based materials, enhancing their melt strength, hydrophobicity, mechanical properties, and antimicrobial activity. These innovations could expand their application from foams to high-performance flexible packaging [136]. The industrial blowing process often necessitates reinforcing thermoplastic starch (TPS) with materials like PBAT, PLA, or LDPE to enhance resistance during air blowing [137]. While higher starch content improves biodegradability and reduces costs, it may also result in lower mechanical strength and humidity resistance. Proper orientation control can improve tensile strength, elongation at break, and tear resistance [138,139]. At the final stage, it is essential to use a dry air stream to cool the blown material to avoid deformations and guarantee the quality of the final product. Cooling rates significantly affect bubble stability dynamics [140]. Air must be blown at a specific pressure to internally cool the tubular piece, eliminating heat so that the product ends up with a more uniform temperature [141]. Once the extrusion process is finished, the blown tubular must be cut or pre-cut to produce bags heat-sealed at the bottom, either individually or on a reel, depending on convenience. Heat-seal ability is crucial to packaging integrity, influenced by temperature, time, and pressure, with techniques such as bar, pulse, ultrasonic, friction, and dielectric, depending on film and product structure [142]. Final bags must be packaged in appropriate packaging containers with barriers against humidity and/or in controlled storage environments, conserving dry and complete bags. Blow molding offers competitive, high-quality packaging with faster production rates, making it an attractive solution for food applications. In developing regions, the method’s need for precise control over temperature, pressure, and humidity poses significant challenges. High moisture can weaken starch-based films, and the use of costly reinforcements like PBAT or PLA raises expenses. Despite these obstacles, with proper biopolymer formulation and public policies that improve access to equipment, infrastructure, and technology, blow molding could establish a viable packaging industry, offering economic and environmental benefits in resource-limited settings.

### 5.3. Print and Cut

Shaping design, printing, and cutting are pivotal stages in starch bag manufacturing, facilitating the incorporation of customized information and designs. Conventional inks can induce contamination or include non-biodegradable components in the final product as they contain solvents, binders, pigments, and additives [143]. Biodegradable inks are composed of various organic and mineral components, such as vegetable oil esters [144,145], as well as plant, animal, and mineral extracts [146] to obtain colored carotenoids, anthocyanins, betaines, quercetin, chlorophyll, and phycocyanin [147]. The ink must be biodegradable to ensure that the decomposition process is complete, safe, and follows quality standards for food packaging [148], and that it does not generate harmful waste to the environment and health, especially for food packing, where material transfer or migration could occur [147,149]. Inks for food packaging must be additionally considered Generally Recognized as Safe (GRAS) [148].

Cutting will depend on the type of plastic form after the extrusion, either in sheet or tubular form. The most common cuts on the market are pre-cut roll bags, which involve perforating cut lines at regular intervals along the roll of material to facilitate the release of individual bags [150]. In this case, the tubular film is sealed to the ends by punching cut lines on the coil before being placed into separate bags. There is also shirt-type bag cutting, which involves cutting a coil of material into two equal parts, followed by a lateral cut at one end to form the bottom of the bag and a longitudinal cut at the other end to form the sides of the bag [151]. Gusseted cutting involves adding extra wrinkles to the sides of the bag to create additional storage capacity [152], using locally sourced, biodegradable inks derived from natural dyes. This fosters an eco-friendly industry without relying on imported materials. Simplified, locally manufactured machinery tailored to the production scale and demand can make this process more accessible and sustainable. By focusing on local resources and adaptable technology, the packaging industry can meet environmental standards while supporting regional economies.

### 5.4. Characterization and Quality Control

Generally, the typical thickness range for flexible packaging is between 20 and 300 µm; thicker thicknesses generate greater tensile strength [153] and thinner thicknesses result in a propensity to rupture. Sizes can range from small bags for individual items to larger bags for bulk products. The natural color obtained is usually opaque off-white, but this can change using color inks. To guarantee that the material obtained is environmentally sustainable, biodegradation and composability tests are required under specific lab and environmental conditions in which different temperatures, humidity, and l charges are tested [153]. Studies have shown that commercial starch-based bags can decompose between 55 and 100 days [154,155], while some blends of TPS with plastic materials such as LLDPE and an oxo-degradant chemical take around 2 to 24 months [156], depending on their composition. Another critical parameter of starch-based bags is the permeation of gases such as oxygen and water vapor (OTR, WVTR). These tests evaluate the ability of the starch material to allow for the passage of these gases through its structure [5]. Reinforcements in starch bags with nanoparticles reduce the empty spaces between molecular chains; for example, adding chitosan nanoparticles creates tortuous structures for water molecules to diffuse and permeate through the material, reducing permeability rates [157], and reinforcement of up to 40% CMC or 10% de kaolin shows similar results, where the water vapor permeability rate drops due to lower availability of (OH) groups in intramolecular chains [158,159]. Other authors showed that the incorporation of 90% PVA decreased water and oxygen permeability by approximately 50%, significantly improving the barrier [160].

**Figure 3:**
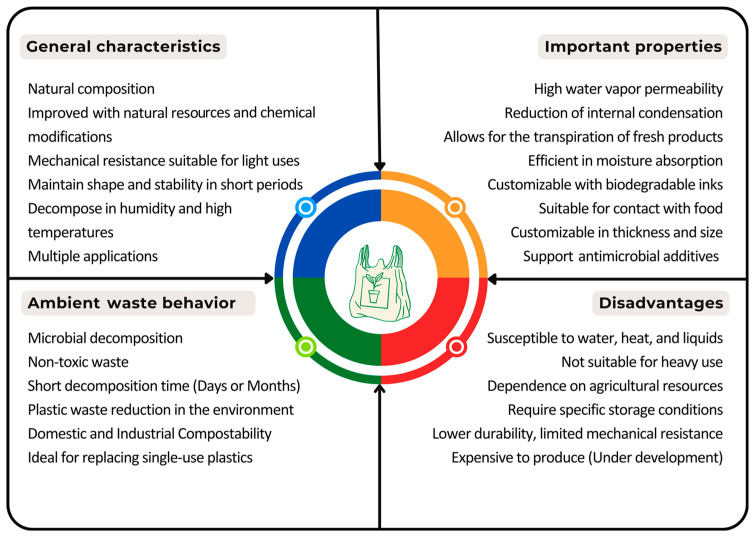
Relevant characteristics, properties, and disadvantages of starch bags.

Migration tests have become relevant for food packaging. This test evaluates the transfer of substances from the bags to the food in contact with them [161]. Analytical techniques are used to detect and quantify the migration of potentially hazardous compounds, such as additives and contaminants that may be contained in the bags [162]. In starch-based bags, the highest migration occurs from glycerol, sorbitol, or similar compounds [163,164,165]. Other tests analyze tear resistance, impact resistance, puncture resistance, dimensional stability, and moisture content, depending on specific application requirements and quality standards. Figure 3 summarizes the relevant and expected characteristics, properties, and disadvantages of starch-based bags.

Specialized equipment ensures the quality and performance of starch-based bags or bioplastics, and critical international standards are strictly followed. The essential equipment includes the Universal Testing Machine (UTM), humidity and temperature-controlled chambers, digital thickness gauge, biodegradation chambers, oxygen and water vapor permeability meters, and the Differential Scanning Calorimeter (DSC). These devices assess parameters such as tensile strength, elongation, elastic modulus, resistance to humidity and temperature variations, thickness uniformity, biodegradability rate, and barrier properties against oxygen and water vapor. Regarding standards, notable ones include ASTM D6400 (compostability of plastics), ASTM D882 (tensile properties), ASTM D5988 (aerobic biodegradation), ASTM D6866 (renewable carbon content), and ASTM E96/E96M (water vapor permeability). Additionally, ISO 17088 harmonizes the specifications for compostable plastics, and the European standard EN 13432 certifies compostability and biodegradation [166].

## 6. Packaging and Storage of Starch-Based Products

Once the production and printing processes are completed, the starch bags must be handled properly to avoid rips or tears during the packing and distribution. As these materials are biodegradable, compostable, and sensitive to changes in humidity and temperature, storage must be in a cool, dry, and well-aired area. Bags must be dry before packing to prevent mold growth and biodeterioration. Starch-based bags should specify the time of expiration for the consumer’s knowledge. A final consideration is the necessity to protect them against rodents and insects during storage as these could become a food source for these organisms. Ensuring proper product rotation is especially important where environmental factors can accelerate degradation. If optimal storage infrastructure is unavailable, cost-effective solutions like airtight containers can be used to protect the material. This reduces waste and ensures that consumers receive high-quality, functional packaging. In contrast, developed countries often have more advanced storage and distribution systems that better preserve these materials. In both contexts, managing product rotation and implementing protective measures are essential for minimizing product loss, maintaining sustainability goals, and optimizing supply chains for biodegradable packaging solutions.

## 7. Final Disposition

In developing countries, alternative and innovative raw materials for biodegradable packing can be classified as industrial or home-composted according to the conditions in which they can decompose. Due to the limitations in infrastructure that developing countries use to process bioplastics that require industrial composting, these countries should develop exclusively home-composted packaging to secure their adequate disposal [167]. Otherwise, local bioplastic production will not effectively address the challenges of solid waste contamination in developing nations. Home-compostable bioplastics decompose within a few months under appropriate conditions, such as moisture and microorganisms, significantly reducing their environmental impact [168]. Moreover, through its biodegradable barrier properties, starch-based packaging reduces food waste by extending the shelf life of perishable products, such as fruits and vegetables. This dual functionality highlights its potential in sustainable food supply chains, particularly in regions with limited waste management infrastructure. Bioplastics from starch can be reused in composting processes, producing by-products like fertilizers or biogas, adding value to waste management [169]. Even if they reach water bodies or soils, their decomposition is faster, and they do not release microplastics that severely affect terrestrial and marine ecosystems. Using non-organic reinforcements, such as nanocomposites or synthetic fibers, significantly improves the bags’ durability and extends their lifespan, allowing them to be reused. However, non-biodegradable additives may interfere with the complete degradation of the bags, posing a moderate environmental threat but on a smaller scale than traditional plastic products that take centuries to decompose. Under optimal composting conditions, thermoplastic starch bags degrade quickly within 1 to 6 months but, if mixed with commercial copolymers or additives, the time is more prolonged and complicated for biodegradation [170].

## 8. Integrated Process Analysis

In recent years, significant advances have been made in developing starch-based biodegradable bags, focusing on improving durability, biodegradability, and utilizing alternative sources. Innovations like patent WO2024091916A2 have enhanced properties by incorporating nanoparticles or fibers during starch gelatinization, improving the homogeneity of the thermoplastic starch matrix and enhancing mechanical properties [171]. Similarly, patent US20240150559A1 adds performance-enhancing additives such as diacids, diglycidyl ether, and silicones, which improve hydrophobicity, reduce glycerin migration, and enhance processability, especially for nonwoven applications [172]. Additionally, patent US6649188B2 introduces a highly flexible films from chemically modified starch give it high mechanical resistance and a good barrier to gases, water, and lipids [173]. These developments offer abundant agricultural by-product alternatives, facilitating cost-effective local production, particularly in developing countries. Technology advances such as reactive extrusion further enable large-scale processing, making starch-based biodegradable bags competitive with traditional plastics in terms of biodegradability, mechanical properties, and cost. Despite the increasing concern about the positive environmental effects of bioplastics and the awareness among consumers, the bioplastic market has not experienced accelerated growth, highlighting the importance of studying and analyzing all the causes associated with it.

**Figure 4:**
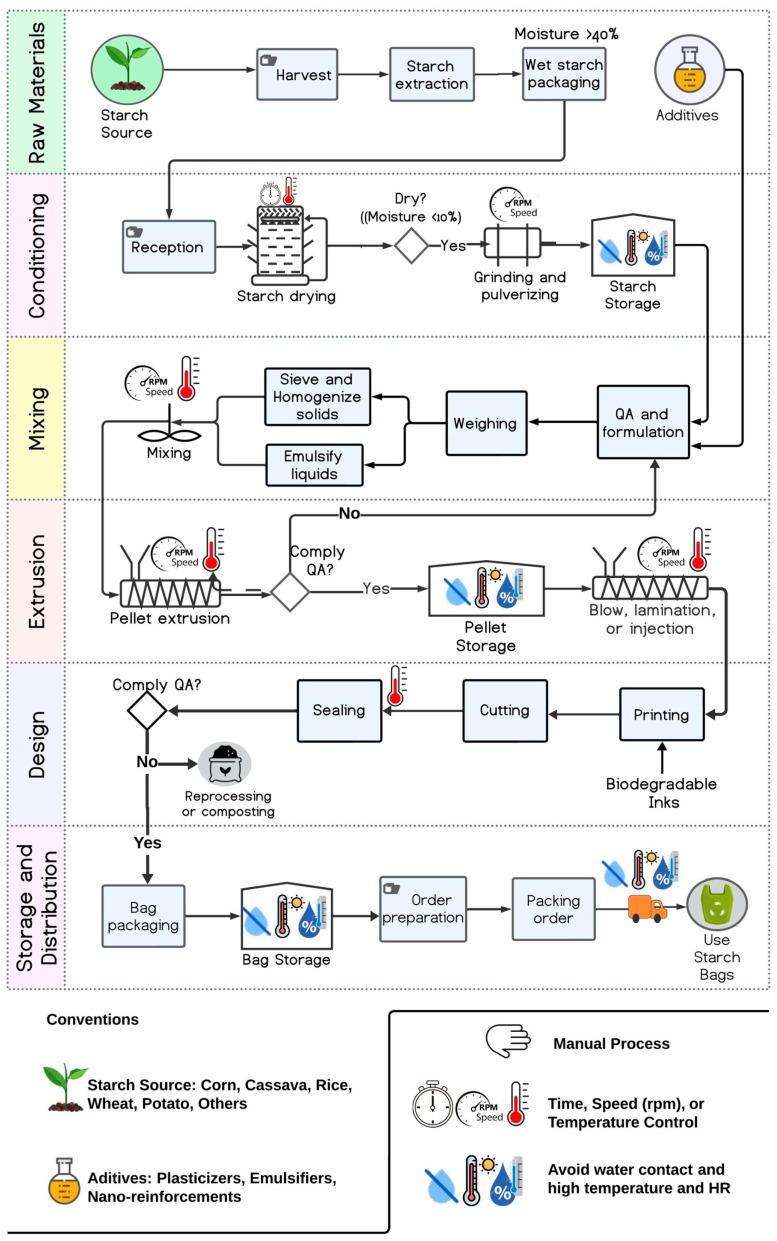
Process flow diagram of the production of flexible starch packaging.

Each stage reviewed confirms that flexible starch-based packaging can be efficiently produced in developing countries, considering the abundance of local raw materials. The complete and integrated process can be systematized through a process flow diagram (PFD) like the one shown in Figure 4. In the integrated process, six key stages can be considered: (1) the extraction and standardization of raw materials, additives, and reinforcing compounds; (2) the conditioning of starch into a dry and dusty material; (3) the mixing and homogenization stage in which each compound contributes to the uniformity and homogeneity of the mixture; (4) extrusion conditions including equipment and energy supply that can be crucial in many developing regions; allowing one to obtain flat or tubular sheets of flexible thicknesses that will enable the construction of multiple types of commonly used bags; (5) bag post-production using cut equipment, access to biodegradable inks, and ink transfer equipment that allows for product customization—waste derived from production can be reprocessed depending on the composition of the biopolymer or disposed of for composting processes; (6) summarizing the final post-production stages and the proper handling of the finished product against environmental factors that may initiate degradability processes before use. The main variables to control during the overall process include temperature and relative humidity to guarantee quality to the final consumer. This price difference may increase depending on industrial development, local availability of raw materials, logistics chains involved, and the supply on the market, which shows the lack of economies of scale in the bioplastics industry in many regions. On a larger scale, the cost of conventional plastics ranges from EUR 1 to EUR 1.5 per kilogram, while starch-based bioplastics are more expensive, ranging from EUR 2.5 to EUR 4 per kilogram [166]. In addition, imported biopolymers such as polylactic acid (PLA) and polybutylene adipate co-terephthalate (PBAT) increase costs, sometimes exceeding USD 5 per kilogram [174]. Despite the environmental benefits of bioplastics, high costs limit their market penetration, with bioplastics accounting for only 2% of global packaging production [175]. As one of the main issues for consumer acceptance of bio packages is the higher cost when compared to petrochemical-based plastics, the price reduction of these manufactured goods is one of the main goals to reach. In developing countries, most of the bioplastics commercialized are imported, increasing the final cost for the consumer. That is why local production constitutes a real alternative for these regions.

The strategy is to reduce imported materials and encourage local industries to produce natural plasticizers and reinforcements. Such value chains could bolster rural economies, creating a circular dynamic where biodiversity resources are sustainably processed into higher-value products, being economically and environmentally sustainable.

It is clear that the offer of packaging called biodegradable is significant, but not all of what it is offered is truly biodegradable and home-compostable, and consumers are not always able to recognize biodegradable packaging at first glance [176]. Bio packaging labels often emphasize biodegradability or use the word biodegradable without clarifying to consumers the degradability limitations of the product or the percentage of non-biodegradable components in it (e.g., oxo-degradable plastic bags) misinforming the consumer about the real environmental impact of the product. More clear and unified labeling should be developed to educate and inform consumers about the products they are purchasing [177].

It has been observed that bio-packaging consumers are willing to use them also for emotional causes [178]. If consumers perceive few benefits associated with the package, negative feelings may arise. Consumer environmental concern is directly related to their packaging purchase intention. But bioplastics cannot be used in the same way as traditional plastics and a false expectation of the consumer when choosing a biodegradable package might discourage them from using them in the future, even knowing all the benefits associated with it. Consumers are required to be educated about how to use and what to expect about bio-packaging for better acceptance. Until now, this is an issue that has not been attended to and that might create a difference in the speed of change in consumption habits. The cost–benefit analysis of starch-based bioplastics indicates that, although the initial costs are higher than those of petrochemical plastics, the long-term benefits justify the investment. In a social development aspect, strengthening local bioplastics industries that use agricultural waste can create jobs in rural areas, contributing to economic growth and poverty reduction. To ensure financial viability, private capital investment requires the adoption of subsidies for machinery, training programs, and research to improve bioplastics’ properties and production processes. Public policies should also encourage the development of circular economies, maximizing local resources and minimizing environmental impact, allowing developing countries to benefit from the economic and ecological advantages without compromising the availability of food sources, overcoming most of the technological barriers described before. Coherent policies, financial incentives, and adequate consumer education about bio-packaging are essential to ensure the viability of this industry around the world and the sustainable implementation of it in regions rich in biodiversity but lacking sufficient infrastructure.

## 9. Conclusions

Producing flexible starch-based packaging in developing countries has the potential to significantly reduce dependence on petrochemical plastics, with environmental and economic benefits. These materials not only reduce food waste by extending the shelf life of perishable goods, such as fruits and vegetables, but also provide sustainable alternatives in regions with limited waste management infrastructure. A sustainable starch supply is crucial, prioritizing non-food sources like agricultural residues or non-edible plants to avoid competition with food resources. This approach promotes a circular economy by converting waste into bioplastics, reducing environmental impact while supporting local economies. Scaling industrial processes like extrusion remain challenging in resource-limited settings, requiring precise humidity and temperature control. Locally built, energy-efficient equipment can lower production costs, reduce import reliance, and foster technological independence. Innovative storage solutions, such as airtight containers or climate-controlled environments, are essential to maintain product quality and minimize waste throughout the supply chain. Public policies, including subsidies, consumer education, and restrictions on single-use plastics, are critical for widespread bioplastic adoption. Additionally, local industries can produce natural plasticizers and reinforcements from agricultural by-products, enhancing durability and reducing dependency on costly imports. Emerging technologies, such as composite techniques and advanced extrusion processes, should be tailored to regional needs to ensure economic viability. Replacing conventional plastic films with starch-based alternatives could reduce CO_2_ emissions per ton of plastic replaced, reducing the carbon footprint of packaged foods. In developed countries, starch-based bioplastics have been successfully used in meal packaging and compostable utensils, demonstrating scalability and consumer acceptance. By addressing these challenges, starch-based packaging can become a sustainable solution that supports global sustainability goals, strengthens local economies, and reduces food system losses.

## Figures and Tables

**Table 1:** Types of starch according to source, composition, and other characteristics.

Starch Source	Ratio Amylose/Amylopectin	Gelatinization Temperature (°C)	Relevant Characteristics	References
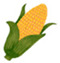 **Corn**	25/75	62–72	A rapid increase in viscosity after gelatinization. Forms an opaque and firm gel.	[42,43]
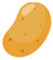 **Potato**	20/80	59–68	Greater water absorption. Lower gelatinization temperature.	[42,44]
** 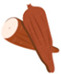 Cassava**	17/83	62–73	Low retrogradation and produces a gel with greater clarity and stability.	[42,45]
** 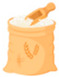 Rice**	19/81	68–78	The texturizing agents used as fat replacements have high extraction costs.	[42,46]
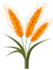 **Wheat**	25/75	58–64	Lower thickening power and gelatinization temperature compared to corn starch. Present large and small granules.	[42,47,48]

**Table 2:** Main additives and reason for use in the manufacture of starch-based bioplastic materials.

Additive	Principal Function	Critical Points	Common Compounds	References
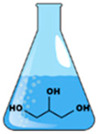 **Plasticizers**	Reduction of intermolecular forces, improved flexibility, decreased fragility and cohesion, reduced shrinkage, increased permeability (gases, solutes, water vapor), balance between cross-linking and flexibility.	Phase separation risk. Increased hygroscopicity, instability, and phase separation at high concentrations. Uncontrolled shrinkage.	Glycerol, Ethylene glycol, Propylene glycol, Sorbitol, Mannitol, Xylitol.	[85,86]
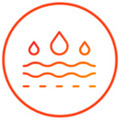 **Emulsifiers**	Optimize the stability and functionality of starch, the dispersion and molecular cohesion of essential oils and fats, and the flexibility of the films. Also improve overall texture and resistance and prevent phase separation.	Emulsifiers affect the films’ water vapor permeability, reduce their tensile strength, and vary in compatibility and stability, affecting the uniformity and dispersion of fatty compounds.	Tween 80, Sodium caseinate, Gelatin, Whey Protein Isolate (WPI), and Whey protein isolated fibril (WPIF).	[87,88,89]
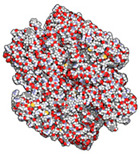 **Cross-linking**	Improves mechanical resistance and thermal stability. Complexity of the process, adjustment of reaction conditions, viscosity reduction, and production of new functional groups. Increase in hydrophobicity and compatibility with hydrophobic polymers.	Some agents are highly toxic and expensive. Cross-linking efficiency varies with pH and requires adjustment of reaction conditions.	Citric acid, boric acid, hydrogen peroxide, glutaraldehyde, phosphoryl chloride, polymalic acid.	[40,90]
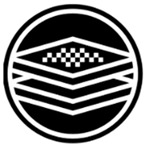 **Anti-block**	Decreases adhesion, creating imperfections on the film surface and preventing full contact between the two layers of the film, reducing blocking.	Alters flexibility, strength, and permeability, affecting processability. Cost and availability must be considered.	Talc, TiO_2_, Calcium, Silica, Kaolin, Calcium Carbonate, Zinc Stearate.	[91,92]
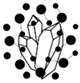 **Nucleating Agents**	Facilitate the formation of crystallization nuclei during the polymer’s cooling. Improve starch’s crystalline structure, increasing its mechanical and thermal properties, such as rigidity, resistance, and thermal stability.	Difficulties in compatibility with starch and homogeneous distribution. Dispersion problems.	Clay nanoparticles.	[93,94,95]
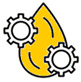 **Lubricants**	Reduce friction, improve processability, and prevent sticking during extrusion and molding. Smoother and more uniform surface, improving the appearance and facilitating the homogeneous distribution of the rest of the additives.	Segregation or incompatibility. Reduce the resistance and rigidity, change optical properties, and increase production costs.	Stearic Acid and Mono and Diglycerides.	[96,97]

**Table 3:** Reinforcement compounds in the manufacture of starch biopolymers.

Reinforcement Compound	Principal Function	Disadvantages	Manufactured Products	References
**Polymers** (PLA, PBAT) 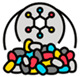	Improve mechanical properties and decrease the biodegradability of starch.	Rigid and brittle compared to other polymers. It is also less available and more expensive since it is obtained through fermentation processes.	Films, packaging, molded products, utensils, injected parts.	[100]
**Cellulose** (Fibers and Pulps) 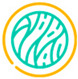	Structural reinforcement and improvement of rigidity.	High moisture absorption. A particular procedure may be required for proper dispersion.	Packaging, bags, biodegradable paper, paper products, foils, molded products.	[101]
**Natural Fibers** (Linen and Hemp) 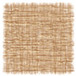	Structural reinforcement.	The range of property improvements depends on the fibers’ source and processing. Proper processing is required for uniform dispersion.	Packaging, reinforced products, biodegradable textiles, injected parts.	[102,103]
**Minerals** (Clays) 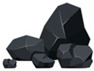	Improve the gas barrier and provide greater mechanical and heat resistance.	Reduce the transparency of products. Higher cost compared to other natural reinforcements.	Packaging, films, coatings, sheets, molded products.	[104,105]
**Fatty Agents** (Waxes, Paraffins) 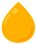	Improve water resistance, improve the water vapor barrier, and increase the lubrication of the material.	Modify the transparency of the products. They can increase migration phenomena and fragility problems.	Waterproof films, packaging, coatings, molded products, and injected parts.	[106]

## Data Availability

The data used to support the findings of this study are available from the corresponding author upon request.
